# Coronavirus: An insight into global research until outbreak of COVID-19 and its implications for the future

**DOI:** 10.7189/jogh.10.020508

**Published:** 2020-12

**Authors:** Doris Klingelhöfer, Markus Braun, Dörthe Brüggmann, David A Groneberg

**Affiliations:** Institute of Occupational, Social and Environmental Medicine, Goethe University Frankfurt, Germany

## Abstract

**Background:**

The currently prevailing global threat of COVID-19 caused the publication numbers on coronaviruses to explode. The awareness of the scientific and public community is enormous. But what about the sense of all these undertakings and what can be learned about the future for a better understanding? These questions were answered with established bibliometric analyses of the time until the avalanche of publications unfolded.

**Methods:**

Chronological, geographical aspects of publication output on coronavirus were also evaluated under the influence of epidemiological and socio-economic parameters.

**Results:**

The trend in publication and citation numbers shows the strong influence of the past pandemics SARS and MERS with an untypical decline afterward. Research is becoming increasingly multidisciplinary over time. The USA and China, as the countries with the highest number of publications, are being displaced by other countries in the consideration of socio-economic and epidemiological aspects, which shows the effect of regional interest in corona research. A significant correlation was found between the number of SARS cases per country and related publications, while no correlation was found for MERS cases and articles.

**Conclusions:**

The results underline the need for sustainable and forward-looking approaches that should not end with the containment of COVID-19.

In line with the aphorism: “Only those who know the past can understand the present and shape the future”, the present study provides background information of research on coronaviruses (CoV) as a basis for the scientific situation of the global pandemic COVID-19 and a source for a better understanding of research patterns from the time before COVID-19.

In 2018, the World Health Organization (WHO) included the coronaviruses SARS-CoV (severe acute respiratory syndrome coronavirus) and MERS-CoV (Middle East respiratory syndrome coronavirus) in the Blueprint list of priority diseases and stressed the need to accelerate research and development because of their “potential to cause public health emergencies of international concern” [1]. Currently, in early 2020, WHO declared the coronavirus disease CoVID-19 the sixth public health treat of international concern [2].

All these pandemic outbreaks caused life-threatening infections in humans because these zoonotic viruses are highly pathogenic. Prior to SARS in 2003, CoV were recognized as pathogens causing only mild infections such as common cold and their clinical significance was not recognized. By 2003, SARS had infected more than 8000 people in 29 countries and killed 774 people. The pandemic ended abruptly, and no cases occurred later [3]. At an animal market in China, the SARS-CoV pandemic was found to be transmitted by palm civet cats [4]. More than 87% of all cases worldwide occurred in China.

The second CoV pandemic MERS (Middle-east respiratory syndrome) occurred in 2012, but unlike SARS, MERS did not end suddenly and cases continue to be recorded until now [5]. MERS affected more than 2400 people and caused 912 deaths in 27 countries by 2019, according to heath authorities worldwide. More than 77% of the MERS cases occurred in Saudi Arabia, transmitted by dromedary camels [6].

Later studies have shown that bats are reservoir hosts for both of these former CoV diseases [7,8].

The current extremely rapid global spread of SARS-CoV-2 has led to the highly dangerous outbreak of the pandemic CoVID-19 with daily increasing numbers of new infections and deaths around the world. At this point in time, the peak of the infections has not yet been reached, so the final figures are not predictable - but it seems that they will reach devastating proportions and will certainly change world societies forever. There are no vaccines yet, and treatment options are still limited. Only symptomatic treatment or support is possible [9]. Due to the enormous spread of infections and the severe course of many cases, the appearance of COVID-19 is accelerating research, which is certainly unique in the history of science. Almost real-time results provide new insights from all areas of science, from basic to applied research. The financing of CoV research is also picking up speed. For the dissemination of this rapidly generated knowledge, international communication is obligatory, and the most common way of disseminating it is the publication of results [10].

Can we learn from previous research patterns regarding CoV? What influence do they have on future research? How can we use past efforts, their intensification and the influences of research on CoV positively to better understand the needs for sustainable and appropriate research? These are compelling questions in light of the currently exploding research output, which, in addition to interesting and meaningful approaches, appears in part excessive, arbitrary and not scientifically sound. Although many scientists around the world are giving their best to solve and improve the pandemic, the current development also gives rise to the pressure to be the first to find the solution and not to be overtaken by colleagues.

Against this background, it is very important to know about previous research. Therefore, we have analyzed the global research output on CoV in the time before COVID-19. In the present study, established bibliometric parameters on chronological and geographical aspects were combined with state-of-the-art visualization techniques based on density equalization principles. Additionally, socio-economic, scientific and epidemiological parameters were related to the publication numbers to obtain an even more meaningful picture of the global landscape of CoV research. The results may help to find more adequate approaches for future research in the financing, planning, implementation and networking of research based on quality and sustainability.

## METHODS

### Methodological platform and data source

The present study belongs to the established bibliometric platform New Quality and Quantity Indices in Science (NewQIS) [11], which examines a broad range of biomedical questions with regard to their translational utility [11-13]. The underlying methodology is constantly evolving and adapted to changing circumstances in terms of global scientific, political and socio-economic characteristics [14]. Standardized bibliometric parameters are combined with newly integrated indices and figures on the research topic in order to analyze and discuss the research landscape appropriately. In combination with state-of-the-art visualization, the results are provided convincingly.

The default database for retrieving the needed metadata for all NewQIS studies is the Core Collection of Web of Science (WoS). Not only because of its status as one of the leading online databases for scientific literature, but also because of its listing requirements, which allow only quality work. Moreover, the provision of the Journal Citation Report (JCR) enables the analysis of citation-based parameters by specifying all citations received for each publication.

### Search term and search strategy

The search was performed at 18/03/2020. To record the respective articles dealing with CoV, the elaboration of an adequate search term is mandatory. The aim is to include as many related entries as possible and to exclude the false entries from the analysis database. Therefore, the term must be a combination of all variants or synonyms of the names of the virus or its transmitted diseases.

In order to find as many entries as possible, the resulting term was: “*corona virus” OR “*coronavirus” OR “SARS” OR “MERS” OR “CoVID-19” OR “severe acute respiratory syndrome” OR “Middle East respiratory syndrome”. WoS offers different modes of search. We applied the title search mode here, because a lot of incorrect entries occur when searching with the topic search mode that also includes abstract and keywords. To further decimate unrelated entries, mostly due to different meanings of the abbreviations SARS and MERS, a specified topic search was added and combined with the Boolean operator “AND” searching for the terms: “virus” OR “epidem*” OR “CoV” OR “Co-V” OR “CoVID-19” OR “patient*” OR “*coronavirus” OR “severe acute respiratory syndrome” OR “Middle East respiratory syndrome”. This strategy ensures content linkage by querying the occurrence of one of these terms either in the abstract or the keywords of the publication. The resulting entries were subsequently filtered by the document type “Articles” to include only original papers in this study.

### Data processing, analyses and visualization

The metadata of the articles found in the manner described above were downloaded and recorded in a database that provides a variety of parameters according to the key information encoded by the WoS tags. Their analyses refer to chronological and geographical parameters, using data on the number of articles in relation to, eg, publication year or country of origin. Other advanced parameters include socio-economic [15-17] and epidemiological data [3,5]. For the epidemiological evaluation, sub-analyses were performed using the same search term, with the only difference that it was reduced to either SARS or MERS.

Furthermore, institutions and research foci were analyzed and the international network was presented. It has to be noticed that the sum of all assigned subject areas of WoS must be higher than the number of articles due to multidisciplinary journals assigned to more than one subject area. In this study an article with more than one subject area assignment to assigned to each area and therefore counts several times.

Citation-based analyses provide information on the recognition of the articles in the scientific community. A threshold was applied to all valuation ratios to reduce distortions of extremely low values, eg, citation rate, and socio-economic ratios. In this way, a concise picture of the global landscape of CoV publications and their development could be created.

The geographical results were partially visualized with density equalizing map projections (DEMP) that are distorted maps according to an algorithm developed by Gastner & Newman [18]. Depending on the value of the evaluation parameter, the countries were either enlarged or reduced in size according to the physical principle of density equalization. Using the VOSviewer application [19], the results of the keyword analysis to determine research priorities are visualized with a network diagram showing clustered nodes and connections for all terms that occurred at least 150 times.

### Methodological limitations and strengths

Every scientific methodology has some limitations that must be reported on. In this case, the first limitation to be mentioned is due to the characteristics of the data source, as WoS does not list all publications in its core collection. WoS requires special recognition for listed journals in order to ensure quality, but this led to the fact that some important articles on CoV could not be included in the analysis as they are not provided by WoS. The title search strategy applied in this study further reduces the database. The advantage, however, is that the included data represent a representative collection that can clearly be used for a valid evaluation. Furthermore, the WoS is said to give preference to English literature, so that the resulting dominance of English-speaking countries is supported by this fact.

Another point that should be mentioned is the manual correction method for metadata on institutions and authors. For this, a threshold had to be introduced to make the procedure feasible. As a result, the exact number of institutions and authors publishing on CoV could not be given. However, the leading institutions and authors could be determined exactly in this way.

Considering the limitations of citation analyses and their impact on meaningfulness in terms of the quality of publications, the use of several citation parameters is appropriate, which gives more weight to the importance of the analysis results for the resonance of the examined publications in the scientific community.

In summary, the applied method has proven to be a valid strategy for the evaluation of bibliometric scientific questions.

## RESULTS

A total of 6905 articles (n) on CoV could be added to the database and form the basis for all analyses except those valid for geographical analyses.

### Research foci

**Figure 1:**
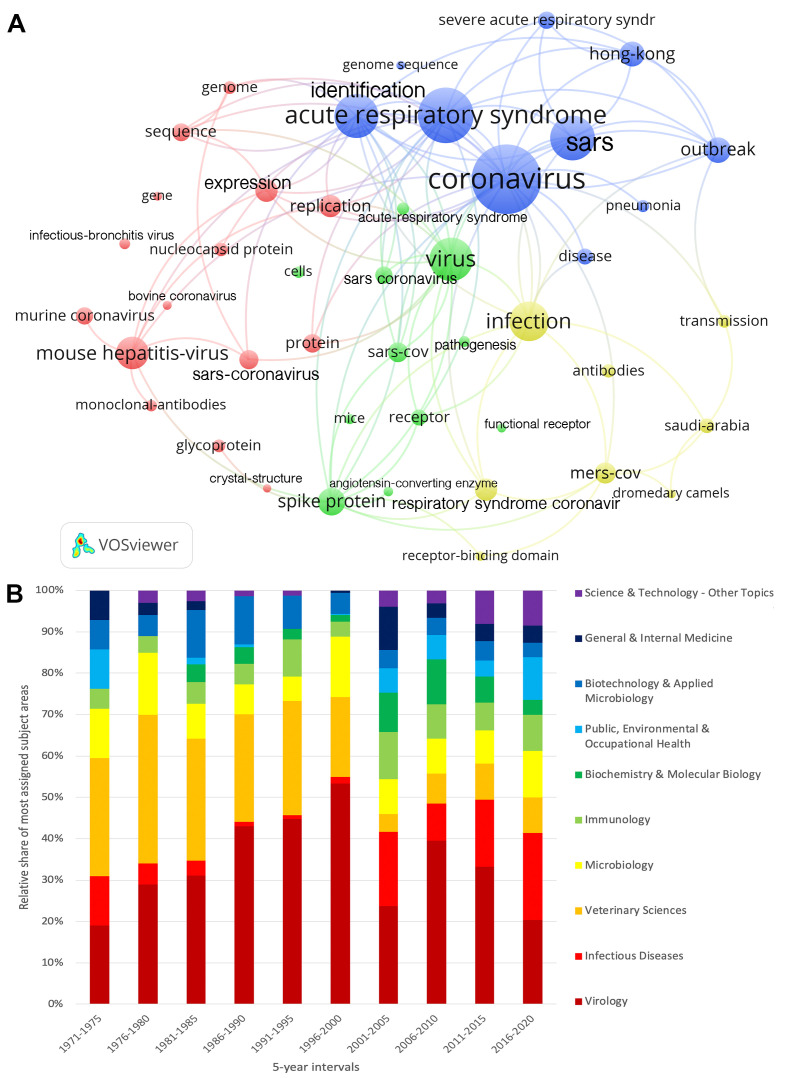
Research foci of CoV publications. Panel A. Keyword clusters with at least 150 occurrences, red = Molecular and biochemical topics, blue = SARS-related topics, yellow = MERS-related topics, green = spike protein and pathogenesis. Panel B. Percentage of most assigned subject areas in 5-year intervals.

Analysis of the keywords used (threshold value: 150 occurrences) revealed four thematic clusters of CoV articles (**Figure 1**, Panel A). First, the molecular and biological topics form a cluster (red). The second cluster (blue) outlines the articles dealing with the SARS epidemic, and the third cluster (yellow) combines the articles dealing with the MERS epidemic. The fourth cluster (green) forms an intermediate group that mainly focuses on the spike protein that is characteristic of CoV, its pathogenesis, and its connection to the other clusters.

In terms of subject areas, the most frequently assigned research fields are *Virology* (n = 2140), *Infectious Diseases* (n = 899), *Veterinary Sciences* (n = 720), *Microbiology* (n = 622), and *Immunology* (n = 558). Looking at the developments over time, it can be seen that the research has become increasingly multidisciplinary (**Figure 1**, Panel B). Especially since the beginning of the 2000s, a change can be observed. *Veterinary Sciences* and *Virology* were relatively declining. The relative frequency of articles assigned to *Infectious Diseases* increased from this time onwards. Other areas of CoV research also became more popular.

### Chronological analyses

**Figure 2:**
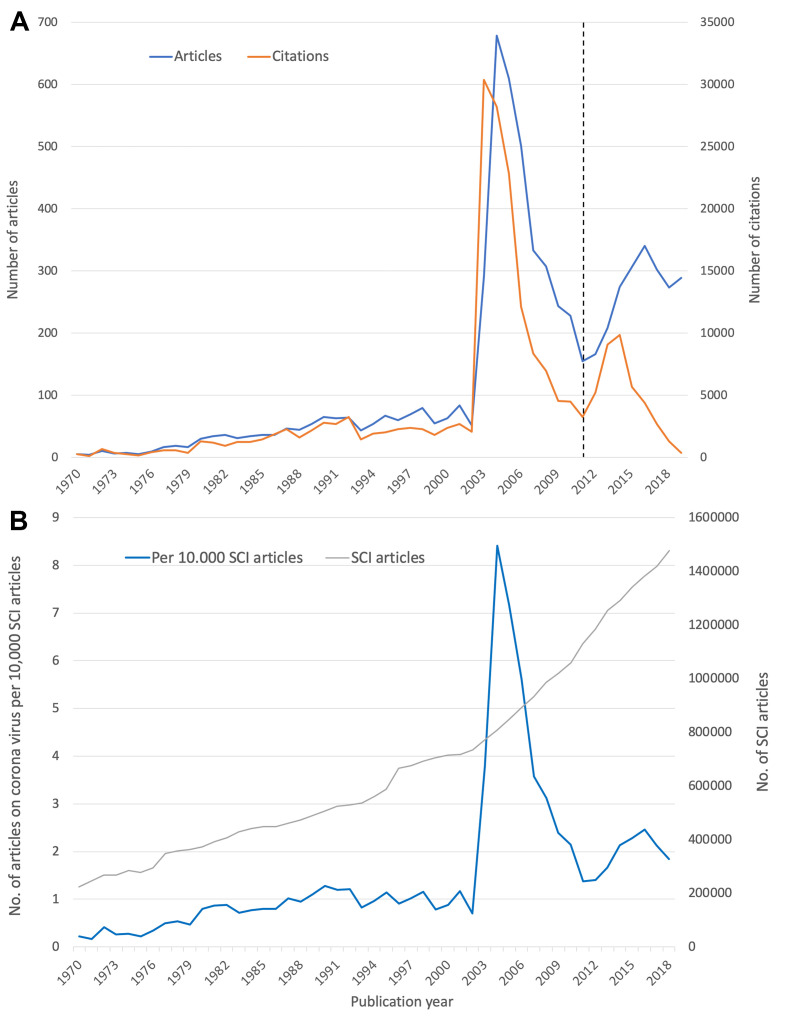
Chronological development of coronavirus articles. Panel A. Number of articles and their number of citations over time, dashed line: cited half-life (CHL). Panel B. Relative number of articles per 10 000 articles indexed in the Science Citation Index (SCI) of Web of Science.

The first article about CoV found in this study was published in 1970. From then on, articles were published every year, but the number remained in double digits until 2003. In this year, the number of articles increased phenomenally to n = 290 articles and then in 2004 to n = 679. The following year was characterized by a sharp decline in annual figures. The second increase in the development of publication numbers on CoV began in 2012 and had its maximum of n = 340 articles in 2016. Then, the number shrank again to 273 articles in 2018 (**Figure 2**, Panel A).

The development of the annual citation frequency showed a similar course, only the peak values were reached in earlier years. The figures remained in the three-digit range until 1979. From then on, they were in four digits until 2003, when they reached the highest value of the entire evaluation period with c = 30 397. After that, the citation numbers decreased until they started to rise again in 2012, reaching the second peak in 2014 with c = 9101.

These courses allow the assumption that the development of CoV articles differs from the overall biomedical development (**Figure 2**B, Panel B). The annual ratio of CoV articles per 10 000 articles listed in the Science Citation Index (SCI) of WoS shows that the share is more or less the same until 2003 with an average ratio of 0.77 CoV articles per 10 000 SCI articles. But this year the development of CoV articles explodes in comparison to the total biomedical publication numbers and reaches a value of more than 8.41 CoV articles per 10 000 SCI articles in 2004. The second highest ratio was reached in 2016 with 2.46 CoV articles per 10 000 SCI articles.

The ranking of the ten most cited articles on the CoV confirms the high recognition of the articles published in 2003 (**Table 1**). Of the ten most cited articles, eight were published in 2003, one in 2012 and one in 2005.

**Table 1:** The most cited articles on coronavirus (CoV)

Authors	Country	Year	c	Title	Journal
Ksiazek, TG et al.	USA, Vietnam, China, Thailand, Singapore, Taiwan	2003	1841	A novel coronavirus associated with severe acute respiratory syndrome	*NEJM*
Drosten, C et al.	Germany, France, Netherlands	2003	1752	Identification of a novel coronavirus in patients with severe acute respiratory syndrome	*NEJM*
Rota, PA et al.	USA, Netherlands, Germany	2003	1490	Characterization of a novel coronavirus associated with severe acute respiratory syndrome	*Science*
Peiris, M et al.	China	2003	1444	Coronavirus as a possible cause of severe acute respiratory syndrome	*Lancet*
Zaki, AM et al.	Netherlands, Saudi Arabia	2012	1303	Isolation of a Novel Coronavirus from a Man with Pneumonia in Saudi Arabia (ev. Erratum 2013)	*NEJM*
Marra, MA et al.	Canada	2003	1275	The genome sequence of the SARS-associated coronavirus	*Science*
Li, WH et al.	USA	2003	977	Angiotensin-converting enzyme 2 is a functional receptor for the SARS coronavirus	*Nature*
Lee, N et al.	China	2003	973	A major outbreak of severe acute respiratory syndrome in Hong Kong	*NEJM*
Guan, Y et al.	China	2003	901	Isolation and characterization of viruses related to the SARS coronavirus from animals in Southern China	*Science*
Li, WD et al.	China, Australia, USA	2005	861	Bats are natural reservoirs of SARS-like coronaviruses	*Science*

c – number of citations, NEJM – *New England Journal of Medicine*

It is obvious that the articles from 2003 deal with the new CoV and the related disease called SARS, which appeared year. Working groups from the USA, China, Southeast Asian countries, Germany, France and the Netherlands identified and characterized the new virus and its association with SARS [20-23]. Canadian scientists worked on the genomic sequence [24].

In 2012, the MERS coronavirus appeared, leading to this year's high-ranking publication of Dutch and Saudi Arabian scientists [25]. The 2005 article, which ranks 10^th^, stated that bats are natural reservoirs for SARS-CoVs [7]. This was a collaboration between China, Australia and the USA.

All ten most frequently cited articles were published in renowned journals. The *New England Journal of Medicine* and *Science* published four of them each. One each was published in *The Lancet* and *Nature*.

### Geographical analyses

The necessary information about the country of origin can be collected through affiliation data. Before 1973 this information is not always available and the gaps are too large to be used. Exactly n = 50 articles could not be assigned to any country of origin, so that n = 6855 articles are included in the geographical evaluations.

**Figure 3:**
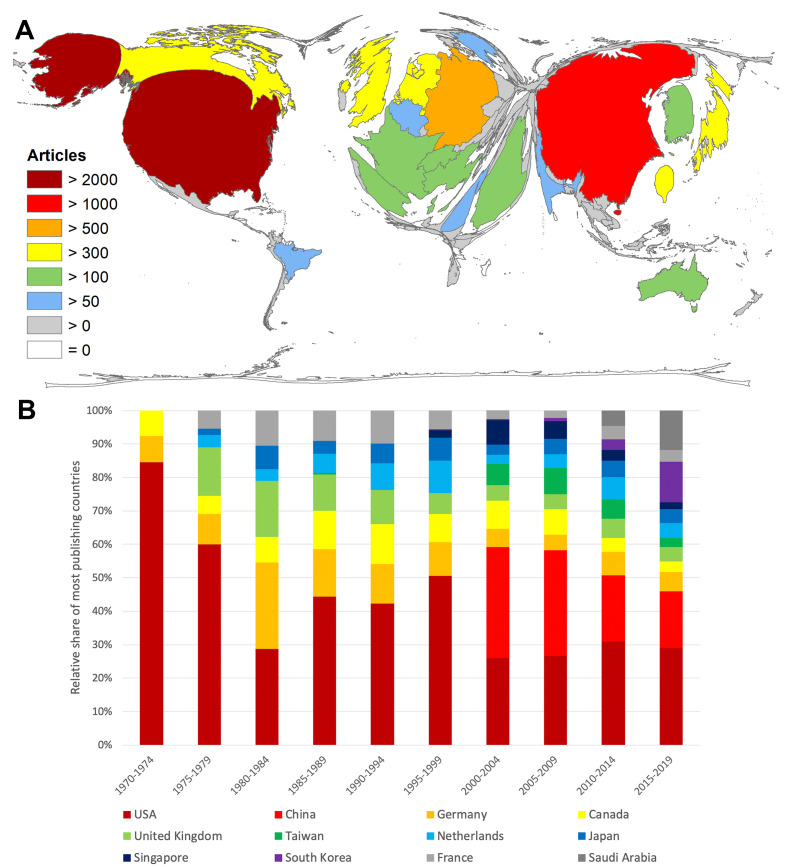
Countries’ publication performance on coronavirus (CoV). Panel A. Number of articles per country. Panel B. Relative share of articles of the most publishing countries in 5-year intervals.

From 1970 onwards, the USA was the country with the highest number of publications for the entire evaluation period (n = 2293), followed by China (n = 1707). By a larger margin, Germany ranked third (n = 505), followed by Canada (n = 488) and the United Kingdom (UK) (n = 413) (**Figure 3****,** Panel A).

The division of the study period into 5-year intervals revealed the beginning of China's research interest at the beginning of the 2000s and Saudi Arabia's interest at the beginning of the 2010s (**Figure 3**, Panel B).

The leading countries were also represented by the leading publishing institutions (**Table 2**).

**Table 2:** Most publishing institutions on coronavirus (CoV)

Institution	Articles	Citations	Citation rate
University Hong Kong, China	398	22141	55.63
Chinese University Hong Kong	217	8846	40.76
CDC, USA	155	8769	56.57
Chinese Academy of Science	139	4079	29.35
University Utrecht, Netherlands	139	7610	54.75
National Taiwan University	132	2534	19.20
University North Carolina, USA	127	5326	41.94
University Iowa; USA	122	3728	30.56
National University Singapore	114	3029	26.57
NIH, USA	106	5617	52.99
University Penn, USA	103	4422	42.93
University Wurzburg, Germany	102	6330	62.06
King Saud University, Saudi Arabia	98	1760	17.96
Erasmus Medical Center, Netherlands	96	11241	117.09
Peking University, China	90	2100	23.33

CDC – Center for Disease Control and Prevention, NIH – National Institutes of Health

**Figure 4:**
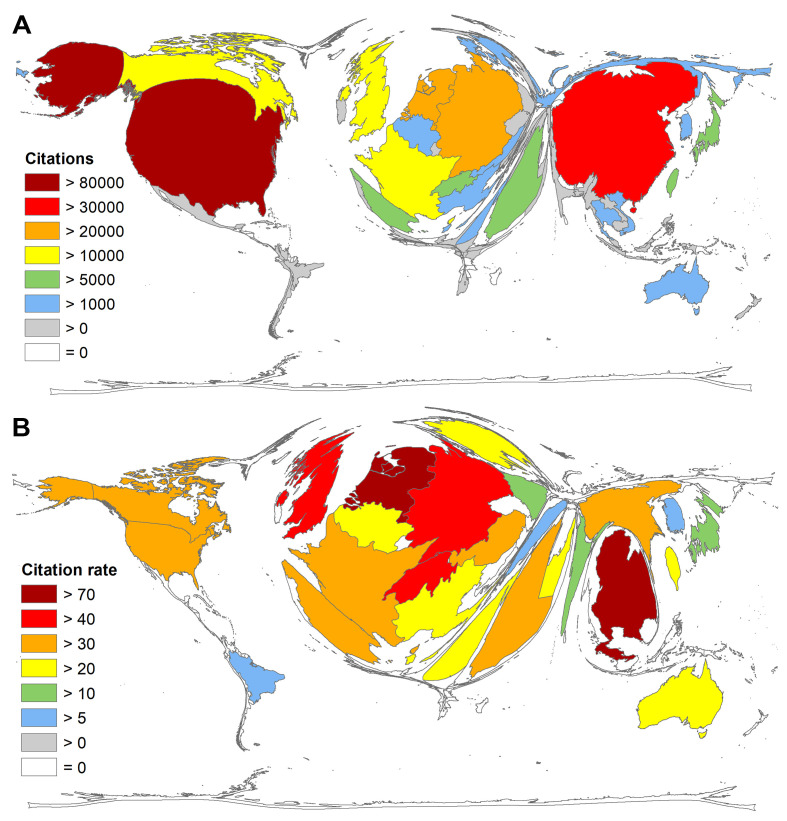
Citation parameters of coronavirus (CoV) articles. Panel A. Number of citations per country. Panel B. Average citation rate (number of citations / number of articles) per country, threshold = 30 articles on CoV.

In terms of citation numbers (c), the landscape looks similar to that of article numbers (**Figure 4****,** Panel A). The list of the five leading countries is the same, with the exception of the Netherlands, which was in third place: the USA (c = 85 381), China (c = 54 907), the Netherlands (c = 25 916), Germany (c = 24 543) and the United Kingdom (c = 18 530). Canada followed in sixth place with c = 17 081.

Looking at the relation of the number of citations to the number of articles (cr = citation rate) of countries with at least 30 articles on CoV, the order shifts (**Figure 4**, Panel B). Thailand was the leading country in this respect (cr = 75.47), although the number of articles with n = 38 only just exceeded the set threshold. Rank second was occupied by the Netherlands (cr = 71.20), followed by Germany (cr = 48.50), the UK (cr = 44.87), and Switzerland (cr = 41.00).

### Inclusion of socio-economic parameters

**Figure 5:**
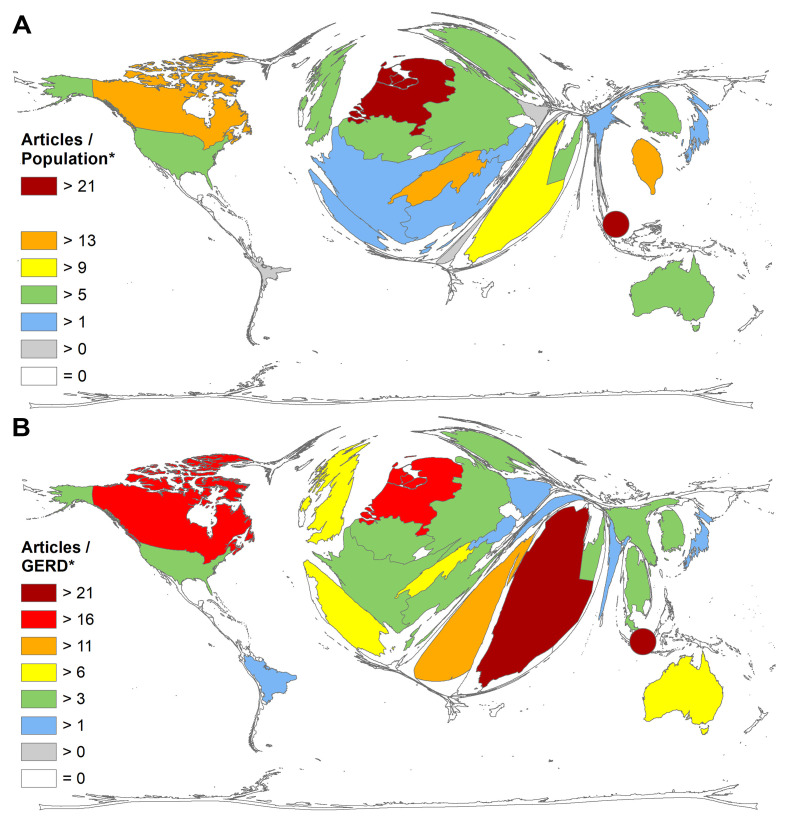
Demographic and science-related parameters of coronavirus (CoV) articles, threshold = 30 articles on CoV. Panel A. Number of articles / population in mill. inhabitants. Panel B. Number of articles / GERD in bn PPP$, GERD = gross expenditure in research and development, PPP$ = purchasing power parity in US-Dollars. * Singapore was the leading country in both analyses, but could not be presented in a distorted way due to methodological limitations. Therefore, it was added manually as point.

When looking at the world map when socio-economic parameters are included in the analysis, the distortion of country sizes shifts again. Regarding the ratio of the number of articles and population size in million inhabitants (RPOP) of countries with at least 30 articles on CoV (threshold) the ranking is as follows (**Figure 5****,** Panel A): Singapore (RPOP = 51.19), the Netherlands (RPOP = 21.39), Taiwan (RPOP = 16.02), Switzerland (RPOP = 15.16), and Canada (RPOP = 13.80).

Another socio-economic parameter, the relation of the number of articles on CoV to the Gross Domestic Product (GDP) in 1000 billion US-Dollars (RGDP) for countries with at least 30 articles (threshold), shows a similar ranking. The rankings of both socio-economic analyses were compromised in **Table 3**.

**Table 3:** Rankings of science-related parameters for countries with at least 30 articles on coronavirus (CoV) (threshold)

Country	Articles	Population in million	RPOP	Rank RPOP	GDP in 1000 bn US$	RGDP	Rank RGDP
Singapore	296	5.78	51.20	HI1	0.49	607.93	HI1
Netherlands	364	17.02	21.39	HI2	0.87	420.37	HI2
Taiwan	376	23.46	16.02	HI3	1.13	334.22	HI3
Switzerland	124	8.18	15.16	HI4	0.49	250.86	HI5
Canada	488	35.36	13.80	HI5	1.67	291.52	HI4
Saudi Arabia	275	28.16	9.77	HI6	1.73	158.87	HI6
Sweden	71	9.88	7.19	HI7	0.50	142.54	HI9
USA	2293	324.00	7.08	HI8	18.56	123.55	HI13
Australia	154	22.99	6.70	HI9	1.19	129.52	HI11
UK	413	64.43	6.41	HI10	2.79	148.13	HI8
Germany	506	80.72	6.27	HI11	3.98	127.17	HI12
Belgium	67	11.41	5.87	HI12	0.51	131.73	HI10
South Korea	291	50.92	5.71	HI13	1.93	150.86	HI7
UAE	32	5.93	5.40	HI14	0.67	47.96	HI19
France	275	66.84	4.11	HI15	2.74	100.47	HI14
Austria	32	8.71	3.67	HI16	0.42	76.94	HI16
Spain	167	48.56	3.44	HI17	1.69	98.82	HI15
Italy	166	62.01	2.68	HI18	2.22	74.74	HI17
Japan	334	126.70	2.64	HI19	4.93	67.72	HI18
China	1707	1373.54	1.24	UMI1	21.27	80.25	UMI1
Egypt	82	94.67	0.87	LMI1	1.11	74.21	LMI1
Poland	33	38.52	0.86	HI20	1.05	31.37	HI20
Thailand	38	68.20	0.56	UMI2	1.16	32.73	UMI2
Turkey	30	80.27	0.37	UMI3	1.67	17.96	UMI4
Brazil	75	205.82	0.36	UMI4	3.14	23.92	UMI3
India	63	1266.88	0.05	LMI2	8.72	7.22	LMI2

n – number of articles, RPOP – number of articles / population in mill. inhabitants, RGDP – number of articles / gross domestic product in 1000 billion US$, UK – United Kingdom, UAE – United Arab Emirates, USA – United States of America, ranking according to RPOP, HI – high-income country, UMI – upper-middle-income country, LMI – lower-middle-income country [26]

The inclusion of parameters referring to the research infrastructure of the countries considers two values. First, the gross expenditure for research and development (GERD) in billion PPP$ (purchasing power parity in US-Dollars) and the number of researchers in million FTE (full time equivalents). Again, the number of articles on CoV for countries with at least 30 articles (threshold) was set in relation to these parameter (RGERD, RRES).

The ranking of RGERD (**Figure 5****,** Panel B) placed also Singapore first (RGERD = 26.63), followed by Saudi Arabia (RGERD = 21.98), the Netherlands (RGERD = 20.22), Canada (RGERD = 17.95), and Egypt (RGERD = 11.98). The second ratio in terms of the number of researchers showed the following countries leading: Singapore (RRES = 8072.96), the Netherlands (RRES = 4267.29), Canada (RRES = 3145.79), Switzerland (RRES = 2834.93), and the USA (RRES = 1627.15) (**Table 4**).

**Table 4:** Rankings of science-related parameters for countries with at least 30 articles on coronavirus (CoV) (threshold)

Country	Articles	GERD in billion PPP$	RGERD	RANK RGERD	Researchers (FTE) in million	RRES	Rank RRES
Singapore	296	11.11	26.63	HI1	0.04	8072.96	HI1
Saudi Arabia	275	12.51	21.98	HI2	0.00	0.00	HI18
Netherlands	364	18.01	20.22	HI3	0.09	4267.29	HI2
Canada	488	27.18	17.95	HI4	0.16	3145.79	HI3
Egypt	82	6.85	11.98	LMI1	0.07	1255.72	LMI1
UK	413	47.81	8.64	HI5	0.29	1425.74	HI7
Spain	167	21.37	7.81	HI6	0.13	1253.80	HI8
Australia	154	21.20	7.26	HI7	0.00	0.00	HI19
Switzerland	124	17.79	6.97	HI8	0.04	2834.93	HI4
Italy	166	32.47	5.11	HI9	0.14	1218.76	HI10
UAE	32	6.52	4.91	HI10	0.02	1434.46	HI6
Belgium	67	14.18	4.73	HI11	0.06	1186.19	HI11
France	275	62.95	4.37	HI12	0.29	952.95	HI12
Sweden	71	16.74	4.24	HI13	0.08	943.56	HI13
USA	2293	543.25	4.22	HI14	1.37	1672.15	HI5
Thailand	38	9.11	4.17	UMI1	0.08	455.91	UMI2
Germany	506	127.11	3.98	HI15	0.41	1223.58	HI9
China	1707	495.98	3.44	UMI2	1.74	980.79	UMI1
South Korea	291	89.83	3.24	HI16	0.38	759.59	HI14
Poland	33	11.44	2.88	HI17	0.10	341.98	HI17
Austria	32	14.58	2.19	HI18	0.04	712.17	HI15
Japan	334	175.84	1.90	HI19	0.68	493.87	HI16
Brazil	75	39.90	1.88	UMI3	0.18	416.69	UMI3
Turkey	30	20.58	1.46	UMI4	0.11	268.11	UMI4
India	63	49.75	1.27	LMI2	0.28	222.62	LMI2

n – number of articles, RGERD – number of articles / GERD in billion PPP$, GERD – gross expenditure in research and development, PPP$ - purchasing power parity in US$, RRES – number of articles / number of researchers in FTE (full time equivalent), UK – United Kingdom, UAE - United Arab Emirates, USA – United States of America, ranking according to RGERD, data on Taiwan was not available, HI – high-income country, UMI – upper-middle-income country, LMI – lower-middle-income country [26]

The socio-economic parameters GDP, GERD, and number of researchers were significantly correlated with the number of CoV articles (*P* < 0.0001), with correlation coefficients (Spearman r) ranging from 0.61 to 0.79.

### Inclusion of epidemiological parameters

In order to show the publication performance of CoV-affected countries in relation to the SARS and MERS epidemics, the relationship of articles on SARS or MERS to the respective cases per country was analyzed. According to WHO [3] 8096 SARS cases occurred until the 2004 epidemic came to an abrupt halt. The sub-analysis of the present study resulted in 3039 SARS-related articles corresponding to the affected countries. **Table 5** summarizes the figures of the analysis. South-Asian countries had the highest percentages of SARS related articles. The correlation of the number of SARS cases per country and their associated publications is with *P* = 0.001 significant.

**Table 5:** Number of SARS cases reported worldwide [3], SARS related articles, ratio of SARS articles per SARS case, and percentage of SARS related articles in the total amount of articles on coronavirus (CoV) per country*

Country	SARS cases	SARS Articles	SARS articles / SARS cases	SARS articles (% of CoV articles)
China	7083	1309	0.18	76.68
Taiwan	346	310	0.90	82.45
Canada	251	270	1.08	55.33
Singapore	238	217	0.91	73.31
Vietnam	63	24	0.38	82.76
USA	27	831	30.78	36.24
Philippines	14	3	0.21	37.50
Germany	9	161	17.89	31.82
Thailand	9	14	1.56	36.84
Mongolia	9	0	0.00	0.00
France	7	72	10.29	26.18
Australia	6	78	13.00	50.65
Sweden	5	14	2.80	19.72
Malaysia	5	2	0.40	12.50
UK	4	119	29.75	28.81
Italy	4	46	11.50	27.71
South Korea	3	58	19.33	19.93
India	3	34	11.33	53.97
Indonesia	2	0	0.00	0.00
Romania	1	0	0.00	0.00
Kuwait	1	0	0.00	0.00
South Africa	1	2	2.00	10.53
New Zealand	1	3	3.00	21.43
Ireland	1	4	4.00	40.00
Russia	1	8	8.00	33.33
Switzerland	1	47	47.00	37.90
Spain	1	50	50.00	29.94

*The sum of the SARS related articles per country is higher than the absolute number of SARS related articles, because international collaboration articles count for every partner country.

**Figure 6:**
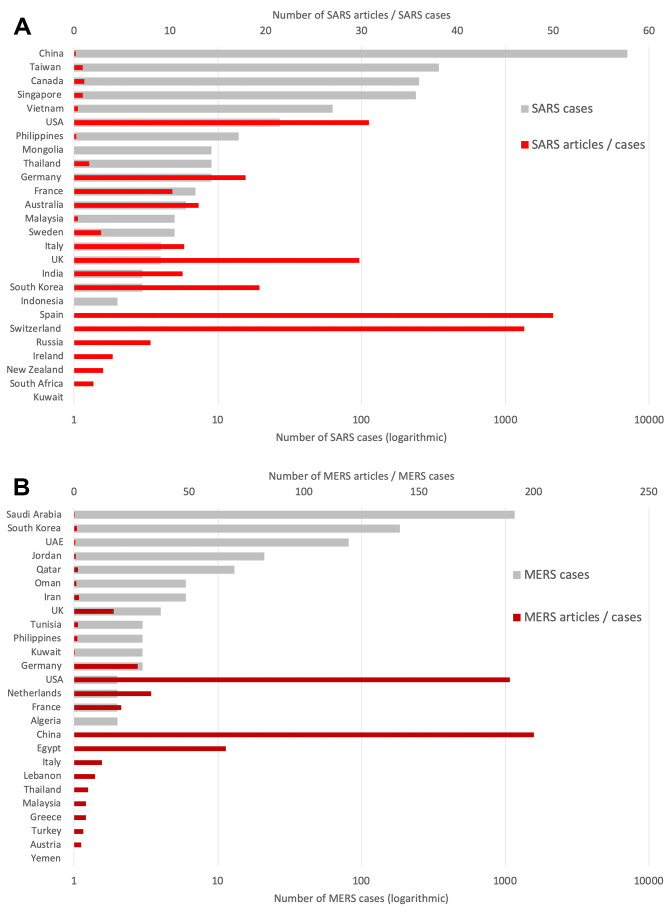
Epidemiological parameters sorted by cases. Panel A. Number of SARS cases (logarithmic display) and number of articles / number of SARS cases. Panel B. Number of MERs cases (logarithmic display) and number of articles / number of MERS cases. UAE – United Arab Emirates, UK – United Kingdom, USA – United States of America

The ratio of SARS related articles and SARS (RSARS) cases showed Spain as country with the highest value (RSARS = 50), followed by Switzerland (RSARS = 47), the USA (RSARS = 30.78), the UK (RSARS = 29.75), and South Korea (RSARS = 19.33). China as the most affected country regarding SARS had a RSARS value of 0.18 (**Figure 6****,** Panel A).

In total, the WHO reported 1511 cases of MERS worldwide occurring with smaller boosts until today [5]. 1068 articles could be clearly assigned to MERS. **Table 6** provides the epidemiological values related to MERS. The affected Middle-East countries had the highest proportion of MERS related articles in their overall CoV articles. The correlation between MERS cases and MERS related articles is not significant (*P* = 0.054).

**Table 6:** Number of MERS cases reported worldwide [5], MERS related articles, ratio of MERS articles per MERS case, and percentage of MERS related articles in the total amount of articles on coronavirus (CoV) per country*

Country	MERS cases	MERS Articles	MERS articles/MERS cases	MERS articles (% of CoV articles)
Saudi Arabia	1165	250	0.21	90.91
South Korea	185	202	1.09	69.42
UAE	81	30	0.37	93.75
Jordan	21	14	0.67	82.35
Qatar	13	20	1.54	90.91
Iran	6	12	2.00	60.00
Oman	6	6	1.00	100.00
UK	4	69	17.25	16.71
Germany	3	83	27.67	16.40
Kuwait	3	1	0.33	25.00
Philippines	3	4	1.33	50.00
Tunisia	3	5	1.67	83.33
Algeria	2	0	0.00	0.00
France	2	41	20.50	14.91
Netherlands	2	67	33.50	18.41
USA	2	379	189.50	16.53
Yemen	1	0	0.00	0.00
Austria	1	3	3.00	9.38
Turkey	1	4	4.00	13.33
Greece	1	5	5.00	31.25
Malaysia	1	5	5.00	31.25
Thailand	1	6	6.00	15.79
Lebanon	1	9	9.00	100.00
Italy	1	12	12.00	7.23
Egypt	1	66	66.00	80.49
China	1	200	200.00	11.72

MERS – Middle East Respiratory Syndrome

*The sum of the MERS related articles per country is higher than the absolute number of MERS related articles, which is due to the fact that international collaboration articles count for every partner country.

The ranking of RMERS (number of MERS articles / number of MERS cases) showed the following order of the five leading countries: China (RMERS = 200), the USA (RMERS = 198.50), Egypt (RMERS = 66), the Netherlands (RMERS = 33.50), and Germany (RMERS = 27.67) (**Figure 6****,** Panel B).

### International networking

Of the total number of n = 6855 articles for the geographical analyses, n = 1716 articles were prepared in international cooperation. The maximum annual international partnerships for CoV research took place in 2004 (n = 135, first publication peak) and in 2016 (n = 126, second publication peak) (**Figure 1**, Panel A). In principle, however, an upward trend can be observed.

**Figure 7:**
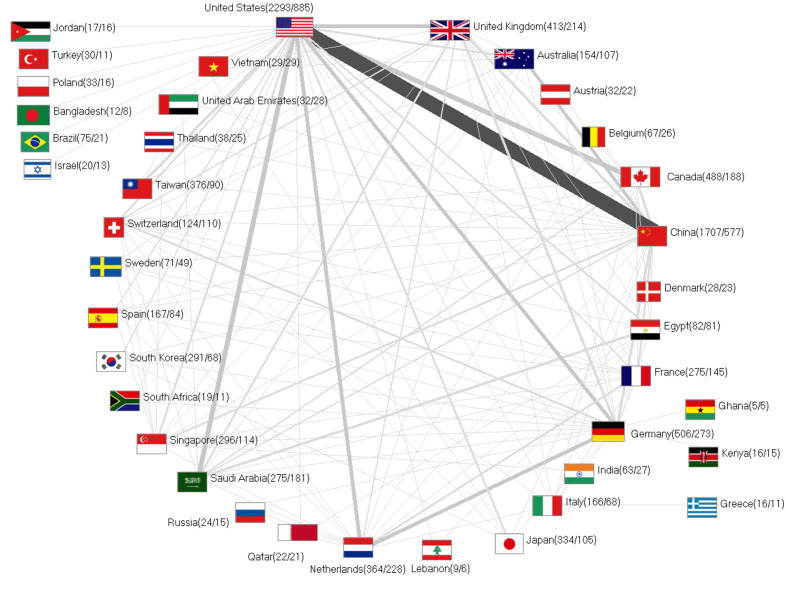
International publication network on coronavirus (CoV) (threshold for the display: at least 5 collaboration articles between countries).

The USA was at the center of the international network and was involved in the five strongest international collaborations (**Figure 7**). The most productive bilateral cooperation in CoV research was between the USA and China with n = 290 cooperation articles, followed by USA/Canada (n = 113), USA – UK (n = 77) and USA - Netherlands (n = 75). The non-US partnership with the highest publication volume was between the Netherlands and Germany (n = 74).

## DISCUSSION

Especially since the beginning of the COVID-19 pandemic, which is causing many serious cases and deaths in many countries, the importance of research on CoV has become clear. Basic research to date, the identification of novel viruses of the two pandemics SARS and MERS, has influenced global research efforts. As more is learned about the background, incentives and impact of research, the impact of more focused and improved focus, planning, implementation, collaboration and communication of scientific projects becomes clearer, for all parties involved.

This is also shown by the identified research foci, which were analyzed by the keywords used in the articles on CoV. However, the proportion of human-related research has increased significantly since the outbreak of SARS, and multi-disciplinarity has also become increasingly widespread over time. The currently rampant COVID-19 disease and its impact on economic, political and social spheres will provide new impetus for CoV research in other scientific fields in the future.

### Chronological influences

The development of publication output on the CoV clearly followed a different trend than that of other biomedical topics in general, which usually increase exponentially over the evaluation period [27] and could be shown in other studies on viral diseases [12,13]. Instead, the increase in the number of articles and citations corresponds to the occurrence of the two pandemics SARS and MERS in the past with peaks at the beginning of each.

In 2003, the year in which SARS emerged, publication numbers rose at an unprecedented rate in science, with an increase of about 500% within one year. Chiu et al. also noted in a 2004 bibliometric study on SARS publications that the high publication rate at the beginning of the pandemic resulted in high citation rates due to the immediate recognition in the scientific community [28]. Now, 16 years later, the present study confirms this extremely high recognition by the scientific community at the time of the SARS outbreak, which even shows a number of citations per publication year, even before the maximum peak in publication numbers is reached.

In 2003, the highest citation rate was achieved with cr = 104.83, which means that each article on the CoV was cited about 104 times on average. This is certainly an extraordinary result. Almost at the same pace, however, the number of publications and citations decreases thereafter, resulting in a value that is below the average biomedical topic. This shows the decline of the scientific interest with the abrupt end of SARS. The awareness of the importance of CoV research as a preventive measure for future virus infections does not seem to have been present at this time. The progression curves of both publication and citation numbers showed a minimum level at exactly the time when the cited half-life (CHL) of publications in biomedical publications is generally reached. Basically, the CHL is given as 7-8 years that a publication needs to reach half of the expected citations, resulting in a maximum peak of citations up to that point [29]. The outbreak of MERS in 2012 caused a second increase, but this time it reached only about one third of the SARS-related figures, due to the lower incidence rates compared to the SARS pandemics. It also showed a declining trend, but only four years after the outbreak. The longer period during which MERS cases occurred also led to a longer lasting interest, but at a significantly lower level. The timeframe of the evaluation was set until March 2020, so that the first months of COVID-19 were included in the analysis. The expected exponentially increasing numbers could even be shown in this small time-interval by a further significant increase in the number of publications.

The short-term effect of research efforts of earlier CoV research and the strengthening of international networking could also be shown with regard to another emerging virus epidemic that occurred in South America in 2015: the Zika virus infection. The publication patterns corresponded to those reported here and showed an unsustainable short-term effect of national and international efforts [30]. This similar publication pattern was reinforced by the enormous research incentives of short-term funding and public recognition due to the acute threat. This is also evident in the case of CoV research, and the same influences must be considered in future approaches.

The most frequently cited publications highlight the outstanding impact of the two pandemics and the years of their outbreaks. Most of the ten articles dealing with the identification and characterization of the novel SARS virus were published in 2003, which shows the importance of the first articles on SARS in the scientific community. One article deals with the isolation of the MERS virus, which was published in 2012, and another with the function of bats as natural reservoirs. These articles were from the countries that are considered to be the most publishing countries throughout the assessment period.

### Geographical influences

What is also true for the publication output of most life science and biomedical topics is the leading position of the USA in terms of the absolute number of publications. The rapid catch-up trend and the now high numbers of Chinese scientists is also not unique for CoV research.

Other bibliometric studies confirmed the ranking of the leading countries in terms of absolute publication figures. Bonilla-Aldana et al. found the USA publishing 34.9%, China 22.4%, and Germany 6.8%. Saudi Arabia, as the country most affected by MERS, contributed only 3.6% to CoV publications [31]. In comparison, the percentages revealed in our study were similar: the USA (33.45%), China (24.90%), Germany (7.37%), and Saudi Arabia (4.01%).

In 2011, Kostoff et al. found in another bibliometric study on SARS the declining share of Chinese and the increasing trend of US-American studies. They stated a higher percentage of highly cited publication authored by China compared to other research fields [32]. These findings have been confirmed by the present results showing also the percentage decrease of Chinese articles. Although, a relative downwards trend of US-American articles could also be shown. This is due not only to the decline in absolute figures but also to the efforts of an increasing number of countries worldwide. COVID-19 will certainly continue to influence this trend enormously. Although the study by Chiu et al. showed a low level of international collaboration shortly after the outbreak of the SARS pandemic in 2004 [28], the pace at which international collaboration on CoV was established thereafter was remarkable [32] and will certainly continue.

As third most publishing country, Germany has been involved in CoV research from the very beginning in the 1970s, when only the USA and Canada was interested in this topic. Although Germany had its highest share at the beginning of the 1980s, it has been able to maintain its position among the three leading countries to date. The most cited German article by Drosten et al. from 2003 ranks second in the overall research on CoV and identified a novel coronavirus in a patient with SARS [21]. This was a German-French-Dutch partnership.

The following ranking consists of Canada, the UK, Taiwan, and the Netherlands. All are involved in prominent research on CoV and have sufficient scientific resources and infrastructure not only for research on CoV.

The Netherlands – ranked 7^th^ in absolute numbers –took a leading position when it came to the results of citation-based, socio-economic and science-related parameters. It also participated in three of the most cited articles and thus also in the identification of SARS- and MERS-CoV as a partner country of Germany, the USA, and Saudi Arabia [21,22,25]. With a relatively high proportion of articles in the field of molecular biology, the Dutch scientists contributed the main research on the structure of the spike glycoprotein, eg, they uncovered the three-dimensional structure [33].

Nevertheless, other countries have led the way with respect to the inclusion of socio-economic and science-related. Thailand received the highest citation rate, although with n = 38 it was only slightly above the required threshold of 30 articles. Thailand's participation in the most frequently quoted article is therefore responsible for this high value. The article by Kziazek et al. [20], which demonstrates the etiological role of a novel CoV in SARS, is a joint effort of the SARS Working Group: USA, Vietnam, China, Thailand, Singapore and Taiwan. Scientists from the Center for Disease Control and Prevention (CDC) worked in addition to them from the *International Emerging Infectious Disease Program* in Bangkok (Thailand) as partners in this study. This program was founded by the *Ministry of Public Health* (Thailand) and the CDC (USA) to establish a prevention and detection system that responds to emerging public health threats. Vietnam was also a partner country in this cooperation, but with n = 29 it was just below the threshold and was therefore excluded from the analysis of citation rates. It should nevertheless be mentioned here. Scientists from the Vietnamese Regional Division of WHO were involved in the research.

Singapore, which was also involved in this successful cooperation via the *Singapore General Hospital*, could also be highlighted concerning the analysis of socio-economic and science-related parameters. It received the highest scores in terms of RPOP, RGDP, RGERD, and RRES. Chahour et al. [34] conducted a bibliometric analysis of the first COVID-19 publications, which confirmed the leading position of Singapore in terms of its demographic characteristics. Without applying a threshold, this study ranked Mauritius first in terms of economic strength. We could not find an article from Mauritius until March 2020, and the Chahour study found one publication from Mauritius. This shows how important threshold applied in our study is to avoid overestimating countries with such a low publication output. With 238 SARS cases, Singapore was one of the most affected countries. The resulting scientific interest and the possible in-situ investigation of the cases caused the publication figures to rise at the beginning of the SARS disease and to fall rapidly thereafter. At the time of the COVID 19 outbreak, however, the figures from Singapore rose strongly again. Tan Tock Seng Hospital (TTSH) is at the heart of SARS research in Singapore, a place where 105 secondary cases mainly affected health workers [35].

### Epidemiological influences

The epidemiological impact and the incentive of in-situ research promoted research on both pandemics of the past and showed the clearly centered focus of the publishing countries.

The exploding publication numbers in 2003 and the second peak starting after 2012 are clearly caused by the outbreaks of SARS and MERS. Therefore, we defined both the number of SARS-and MERS-related articles and set them in relation to the number of disease cases of each country.

In the case of SARS, China had clearly the most cases worldwide and had also written the most articles about it. This was followed by the USA and Taiwan. However, the ratio of SARS articles from China and Taiwan to cases was relatively low. In general, the countries with the most cases did not reach the top ranks, as case numbers varied widely among publishing countries, ranging from more than 7000 in China to only one case in eight countries.

Therefore, Spain and Switzerland reached the highest ratios. But with only one identified article, these high rates are not surprising, as both countries were among the 20 most publishing countries. The contribution of literature from both countries to the CoV literature was relatively stable and has fluctuated only slightly since the early 2000s at an average level of about 10 articles per year. Therefore, the publication efforts of both countries could not be directly linked to the outbreak of SARS or MERS.

With regard to MERS, most of the contributions came from the USA, which was also found in a 2016 study [36]. Saudi Arabia produced the second most articles on MERS.

As far as the relationship between MERS publications and MERS cases is concerned, the situation is similar to that of SARS. Saudi Arabia also achieved a relatively low ratio with most MERS cases. Here, the USA and China are the highest-ranking countries, demonstrating their overall interest in CoV research and also focusing on the MERS pandemic, despite the relatively low case numbers. Egypt also came into the focus of this analysis because of the occurrence of only one case and the associated high ratio. However, Egypt's interest is directly related to the outbreak of MERS, as it has seen a significant annual increase in publication numbers from that point on. The occurrence of MERS-CoV in Egyptian dromedary camels and its similarity to the human type influenced the regional research interest [37]. Another causal factor is the strong cooperation partnership with Saudi Arabia and USA in CoV research. In addition, Egypt ranked 5^th^ in the inclusion of GERD as a marker of economic strength and was the only country that did not have a high-income status among the top 15 countries.

The analysis by Chiu et al. from 2004 [28] found no evidence of a correlation between the number of SARS cases and the number of publications in the publishing countries [28], while our results showed a significant correlation. The reason for this discrepancy seems to be the timing of evaluation. In contrast to Chiu et al. who consider the beginning of publication activity on SARS, the present study shows the results of development 16 years after the appearance of the pandemic. During this period, the affected nations seem to have created an awareness of the importance of SARS research and the international interest in CoV research that responds to it.

However, with regard to MERS, our study could not demonstrate a statistical correlation between the number of cases in countries and the number of publications. This could be due to the low number of MERS-related cases and the resulting centered interest.

Taking into account the unusual patterns of previous CoV research, the current situation of COVID-19-related publication output must be evaluated and discussed accordingly. A short note on the most recent publication figures, which include the output of 2020, already shows 3930 publications on the CoV that were found with the same search term (as of 19.05.2020).

Of these publications, only 1230 are articles (31.3%), which means that almost more than two-thirds of the publication includes other types of documents, especially editorial materials and early access, which are almost invisible in the previous period. As a rule, articles make up the largest share of document types. The most frequently assigned subject area in 2020 was *General and Internal Medicine*, which pushed back the fields of *Virology* and *Infectious Diseases* as the most frequently assigned areas of previous research, thus providing an indication of publication in more interdisciplinary journals. The field of *Public, Environmental, and Occupational Medicine* moved on to third place. These figures imply the enormous interest of the scientific community and the enormous willingness to publish. But it leaves one questioning the priority given to quality and shows the attitude of publishers to value the rapid publication of articles related to the CoV. China has the largest share of articles in 2020, followed by the US and Italy. This is due to the advance of Chinese science and the high number of cases in both China and Italy. The results of the present study show the need for sustainable, valid and high-quality research in global cooperation to address not only the current pandemic but also future threats. The increasing divergence of research areas allows for more interdisciplinary approaches, which should be completely free of any blinkered ambitions or dogmatic reservations about the big picture.

## CONCLUSIONS

What concrete effects the current CoVID-19 pandemic will have on the global landscape of CoV research can currently only be estimated. Funding is rising exorbitantly, and the first figures for 2020 show an equally exorbitant increase in publications. As a result, the development of vaccines and effective therapeutic methods can be expected in the near future. But the question arises whether this is a short-term effect which, once CoVID-19 is contained, will lead to an equally sharp decline in publication numbers as was observed with the earlier CoV pandemics SARS and MERS? The need for continued interest and research efforts worldwide is characterized by the characteristics of the past, which lead to a lack of basic knowledge about CoV, about advanced therapies and to difficulties in the search for vaccines.

The present results show the development and incentives for research in the period before CoVID-19 and underpin the need for awareness and sustainable international relations for best possible strategies and the benefit of scientific projects. This is also important in view of the fact that the effects of the prevailing climate change will influence the incidence of zoonotic diseases caused by CoV through human and animal accumulation. This will certainly lead to the need to combat new groups of viruses in the future. Therefore, sustainable and climate-friendly approaches must also be pursued in science, especially if they focus on the newly emerging aspects of CoV research in relation to political and economic issues.
